# Strengthening Actions for Menstrual Health and Hygiene Interventions for Promotion of Women’s Health in Nepal (SAMIP): Protocol for a Participatory Intervention Development Study Using Realist Synthesis, Human-Centered Design, Intervention Mapping, and Arts-Based Methods

**DOI:** 10.2196/89117

**Published:** 2026-04-23

**Authors:** Sara Elizabeth Baumann, Hannah Schwarz, Annika Agarwal, Bhimsen Devkota, Elizabeth Miller, Ada Youk, Sara Parker, Sanjana Murthy, Nikita Sharma, Archana Shrestha, Madhusudan Subedi, Nirajan Khadka, Mary Hawk

**Affiliations:** 1 Department of Behavioral and Community Health Sciences, School of Public Health, University of Pittsburgh, 130 Desoto St, Pittsburgh, PA, 15261, United States, +1 412-383-3526; 2 School of Medicine, University of Pittsburgh, Pittsburgh, PA, United States; 3 Department of Health Education, Tribhuvan University, Kirtipur, Bagmati Province, Nepal; 4 Bikash Shrot Kendra, Kathmandu, Bagmati Province, Nepal; 5 Department of Biostatistics, School of Public Health, University of Pittsburgh, Pittsburgh, PA, United States; 6 Department of Humanities and Social Science, Liverpool John Moores University, Liverpool, England, United Kingdom; 7 Department of Anthropology, Dietrich School of Arts and Sciences, University of Pittsburgh, Pittsburgh, PA, United States; 8 Department of Public Health, School of Medical Sciences, Kathmandu University, Dhulikel, Bagmati Province, Nepal; 9 Department of Community Health Sciences, Patan Academy of Health Sciences, Patan, Bagmati Province, Nepal; 10 Aama Surakshya Nepal, Kathmandu, Nepal

**Keywords:** Nepal, menstrual health, human centered design, intervention mapping, community engaged research, arts-based research, realist synthesis, menstruation, intervention design

## Abstract

**Background:**

Menstrual restrictions remain widespread in Nepal, where approximately 90% of women and girls follow at least one restriction. One of the most harmful practices, chhaupadi (severe menstrual seclusion), requires women and girls to isolate in huts during menstruation and is associated with substantial physical and psychological risks, including injury, assault, smoke inhalation, and social exclusion. Although interventions have attempted to address chhaupadi, effectiveness has varied, and no comprehensive assessment has identified which components work, for whom, and under what conditions. Few prior efforts have meaningfully engaged communities in intervention design to ensure contextual relevance and sustainability.

**Objective:**

This protocol describes a three-aim study designed to (1) elucidate mechanisms underlying the success or failure of chhaupadi interventions; (2) co-design a culturally grounded, theory- and evidence-informed intervention in partnership with communities; and (3) pilot and evaluate the intervention using a controlled trial design.

**Methods:**

This multiphase study integrates realist synthesis, intervention mapping, human-centered design, and arts-based research. In aim 1, we will conduct a realist synthesis of published and gray literature, complemented by knowledge-sharing workshops and expert interviews to identify underlying mechanisms explaining chhaupadi intervention success or failure. In aim 2, synthesis findings will inform participatory intervention development. Using the intervention mapping framework, we will convene a co-design workshop incorporating human-centered design and arts-based research methods (eg, asset mapping, impact ladder, concept posters, and communal storytelling) with women, adolescents, and community collaborators to develop intervention components and implementation strategies. In aim 3, the co-designed intervention will be piloted in a controlled trial comparing intervention communities (full community-designed intervention) with control communities (menstrual health education only). Quantitative measures will assess knowledge, attitudes, and practices related to chhaupadi, self-efficacy, mental health, and reproductive health outcomes. Implementation indicators (eg, enrollment, acceptability, appropriateness, and feasibility) will also be assessed. Qualitative interviews and discussions will examine implementation processes, barriers, facilitators, and contextual influences.

**Results:**

The study was funded in September 2023. Aim 1 activities began in April 2024. To date, 69 materials met the inclusion criteria, and a workshop with 8 organizations and 44 interviews has been conducted. Analysis was completed in February 2026, with results expected in the summer of 2026. Aim 2 co-design workshops began in March 2025; eleven women participated in the workshop, and an additional 123 community members have been consulted. This has resulted in a draft intervention called “Our Voice: Transforming Lives through Ending Chhaupadi.” Aim 2 results are expected in Fall 2026. Aim 3 data collection will begin in Winter 2027.

**Conclusions:**

By systematically identifying effective intervention mechanisms and embedding community leadership throughout design and implementation, this study aims to develop a sustainable strategy to reduce harms associated with chhaupadi and improve women’s and girls health. This integrated framework may also inform interventions addressing other socially embedded health practices in low-resource settings.

## Introduction

### Menstruation Studies and the Nepal Context

Despite being a natural and physiological process [1,2], menstruation is often associated with health and safety challenges, stigma, and human rights concerns, especially in low-resource settings [3]. Guided by the field of critical menstrual studies, addressing health challenges associated with menstruation requires critical engagement with systems of power, socially constructed realities, and lived life course experiences, extending past anatomical explanations to understand how menstruation is interpreted and experienced [4].

In many cultures, menstruation is shrouded in stigma and taboos, leading women to adopt harmful practices [5-12]. In Nepal specifically, menstrual restrictions are widespread; 90% of women and girls follow at least one menstrual restriction [13]. One of these traditions, chhaupadi (severe menstrual seclusion), is a social-religious tradition in which women and girls isolate in menstrual huts or sheds during monthly menstruation (referred to as minor chhau) [14-17]. Many women also isolate to these sheds during the postnatal period (referred to as major chhau or chhau period) [18]. The practice is prevalent in the Karnali and Sudurpashchim provinces of Nepal, where overall development and gender equality are the lowest nationally [16,19]. Chhaupadi was banned by the Supreme Court in 2005 [20], declared a form of gender-based violence in 2010, and criminalized in 2017; however, it prevails in parts of the country [21].

Chhaupadi poses significant health and safety risks for women and families [13,16,17,22]. Physical health risks include snake bites and animal attacks, hypothermia [23], pneumonia [24], suffocation from lighting fires in the sheds to keep warm [14], rape [14], and, in some cases, death [16,17,19,25]. Poor hygiene while practicing chhaupadi puts women at increased risk of infectious disease, as well as infections that can lead to adverse pregnancy outcomes, including preterm delivery [26] and acquisition of sexually transmitted infections [27]. Chhaupadi also impacts the well-being of children who sleep with their mothers or female family members in the sheds; cough, fever, diarrhea, and pneumonia are commonly reported [28]. Women and girls are also psychologically affected during their stays in sheds. Following the practice, which often involves limited security, can leave women in perpetual states of fear [14,29], along with feelings of loneliness [23], stress [30], humiliation [31], isolation [30], and low self-esteem [16]. One study found that 31.4% experienced sadness or depression, and 20% experienced fear of being abused while sleeping in the sheds [31].

Girls who follow chhaupadi have expressed that they follow the tradition due to several deeply held reasons, such as religious and spiritual beliefs, family tradition, fear of negative consequences (eg, potential illness or harm to family if they do not uphold the tradition), and social pressure to maintain the practice [22]. These factors are particularly important to consider when designing interventions.

Chhaupadi sits at the intersection of 2 well-documented bodies of literature: menstrual stigma and harmful traditional practices. Cultural taboos perpetuate menstrual stigma, restrict menstrual hygiene, and hinder efforts to improve knowledge and female well-being; as such, millions of women worldwide face challenges in managing menstruation that negatively affect their health, education, labor force participation, and productivity [32]. Scholars have noted that policy responses alone are often insufficient to dismantle these norms: while public policies can recognize menstrual stigma and set out to break the silence surrounding menstruation, alone, they do not necessarily contribute to dismantling it [33]. Chhaupadi exemplifies this policy-practice gap—criminalized in Nepal since 2017, yet still widely practiced [21]—and thus offers a critical case study in the limits of legislative approaches to gender-based harmful practices.

In the scholarly literature, chhaupadi has been classified as a harmful traditional practice—a term used to describe culturally embedded customs that violate human rights and cause physical, psychological, or social harm, particularly to women and girls [34]. Scholarship on harmful practices in Nepal has documented the wide-ranging health and development consequences of culturally and socially embedded customs targeting women and girls—among them early marriage, dowry, and menstrual seclusion practices such as chhaupadi [12]. While chhaupadi is specific to Nepal, menstrual restrictions and taboos are documented across South Asia and beyond. Menstruation poses particular challenges to young women in India and other South Asian countries due to high levels of menstrual stigma, where menstruating girls and women are often considered dirty or impure and face taboos and restrictions on their mobility, daily activities, and hygiene practices [35]. Chhaupadi thus represents a particularly severe manifestation of a regionally and globally documented phenomenon.

### Efforts to Address Chhaupadi Harms

A range of strategies have been used to address chhaupadi, including awareness raising, education, the physical removal of menstrual sheds, legal sanctions, and the conditional withdrawal of state services [36-38]. Demolition of menstrual sheds has been one structural intervention used by government and NGO actors to eliminate chhaupadi, yet its effectiveness has been limited. In some communities, the removal of sheds has been linked to increased danger for women and girls, who face pressure to practice seclusion regardless of hut destruction, and may resort to sleeping in caves or open spaces without shelter. Local officials have acknowledged that women have rebuilt sheds out of fear, suggesting that physical demolition without attitude change does not constitute a meaningful victory [23,39].

Other intervention approaches that have seen some success are working in partnership with dhamis (traditional healers) and health workers. Interventions that engage traditional healers as agents of change have shown some promise in addressing chhaupadi, reflecting their significant community authority [40]. Other recent studies also suggest that harnessing the expertise of female community health volunteers [41,42] and community health nurses [43] can offer a way to shift attitudes around chhaupadi and associated harms.

Despite these efforts, among others, progress has been modest and often short-lived [38]. The literature points to deeply held beliefs linking the abandonment of menstrual restrictions to illness and misfortune as a significant impediment to change [38]. Effective responses must also account for variation across caste, ethnicity, and religion, as menstrual practices are embedded in complex socio-cultural and religious frameworks that resist one-size-fits-all solutions [13,22,32,41].

### Approach

No known studies to date have comprehensively assessed which components of chhaupadi interventions hold promise. Additionally, no known prior interventions have fully engaged communities in the intervention design process to ensure suitability, sustainability, and centering of community values and voices. Pinpointing salient mechanisms and partnering with communities in crafting solutions is needed for sustainable change, and thus is a central objective of this 5-year study.

We intend to first rigorously review intervention evidence using realist synthesis (RS), a method for reviewing and synthesizing evidence that allows researchers to move beyond whether an intervention is effective, and rather understand the complexities of what works, for whom, and under which circumstances [44,45]. An RS is well-suited to study chhaupadi interventions because it allows for an in-depth examination of how, why, and under what contextual conditions these interventions succeed or fail, capturing the complex social, cultural, and structural factors that shape their effectiveness.

These findings will be used to inform the co-design of a chhaupadi intervention with community members. To facilitate this process, we will harness Intervention Mapping (IM), a systematic and evidence-based framework for planning health promotion interventions [46]. By engaging community members throughout intervention design and gathering feedback via an iterative process, we expect to codevelop a culturally appropriate intervention to pilot test in a clinical trial, ultimately aiming to improve women’s health in Nepal.

This paper describes a protocol for conducting participatory and creative menstrual health intervention research in Nepal that aims to fill gaps in understanding how to sustainably address harms associated with the behavioral practice of chhaupadi (severe menstrual seclusion). This study is innovative and timely, as it is the first study to develop a comprehensive assessment of existing chhaupadi interventions and the first to use a co-designed intervention approach to address the practice. This integrative 3-pillar approach—drawing on IM, human-centered design (HCD), and arts-based research (ABR)—seeks to generate not only a cocreated, theory- and evidence-based intervention, but also an innovative methodological framework adaptable to addressing multifaceted health issues in other low-resource contexts.

### Specific Aims

Three specific aims guide our inquiry:

- Aim 1: Elucidate the mechanisms of success or failure of chhaupadi interventions to date. Via review of scientific and gray literature and key-informant interviews (n=35), we will conduct an RS [45] of extant studies/interventions to identify opportunities and develop a theoretical model to inform chhaupadi intervention design.
- Aim 2: Co-design a chhaupadi intervention with community members. Based on aim 1 findings, we will conduct a 2-week co-design workshop with women and girls who practice chhaupadi (n=10), guided by principles of IM, and using HCD and ABR methods to ultimately develop a culturally relevant chhaupadi intervention. We will elicit feedback on the intervention via community dialogue sessions (n=2, 10 people each), community-level key informant interviews (KIIs) (n=8), and menstrual health expert interviews at the national level (n=8). Additional insights will be gathered via rapid situation analysis in 4 additional chhaupadi practicing communities to understand how the intervention may need to be tailored to specific contexts in future scale-up work (n=75 interviews).
- Aim 3: Pilot the chhaupadi intervention with community members to assess feasibility and acceptability. Two matched communities will be identified and randomly assigned to intervention and control (menstrual education only) at the community level (n=25 intervention, n=25 control). Surveys and focus groups with participants will be conducted to assess feasibility, acceptability, and changes in health attitudes and behaviors.

## Methods

### Study Design

**Figure 1.**
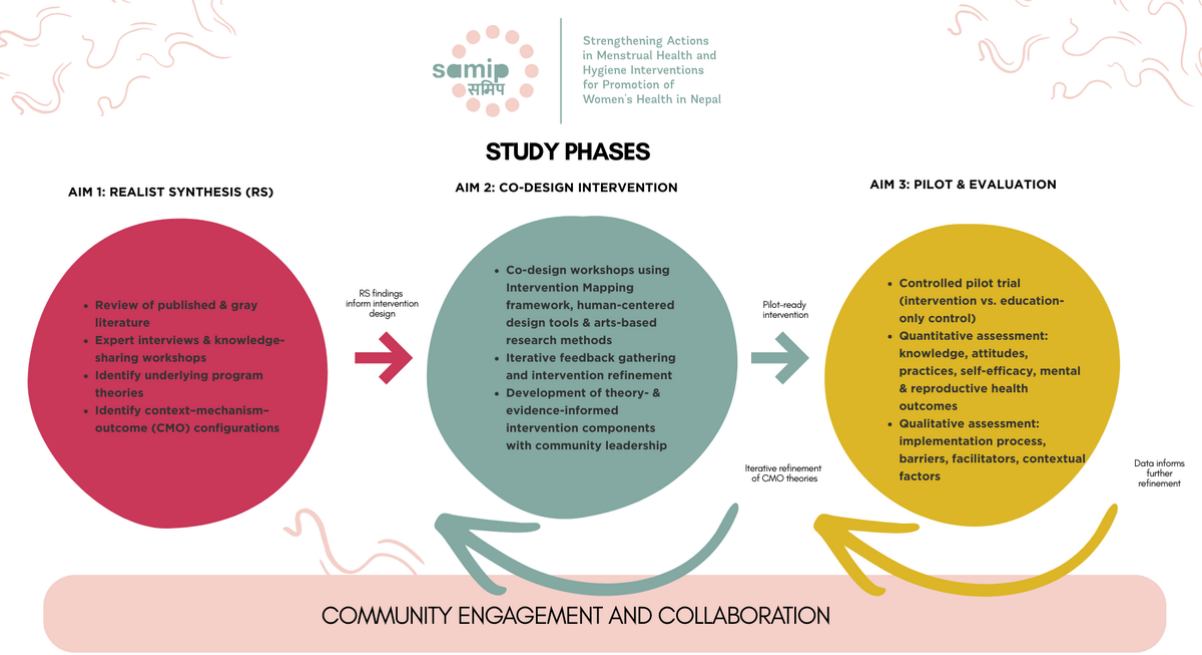
Schematic overview of the SAMIP (Strengthening Actions for Menstrual Health and Hygiene Interventions for Promotion of Women’s Health in Nepal) study workflow. The study is conducted in 3 linked phases. Aim 1: realist synthesis (RS) to identify mechanisms of success and failure of prior chhaupadi interventions. Aim 2: co-design of a community-informed, theory- and evidence-based intervention using intervention mapping (IM), human-centered design (HCD), and arts-based research (ABR) methods. Aim 3: pilot test and evaluation of the intervention in a controlled trial. Iterative feedback loops reflect adaptive refinement of program theories and intervention components throughout the study.

In this 3-phase study called Strengthening Actions for Menstrual Health and Hygiene Interventions for Promotion of Women’s Health in Nepal (SAMIP), we will apply a suite of innovative and tested research methods to address our study objectives (Figure 1). We will conduct an RS (aim 1) to elucidate mechanisms of success or failure of chhaupadi interventions to date, which will inform community-led intervention development using principles of IM, as well as HCD and ABR tools (aim 2). Next, we will pilot the community-designed chhaupadi intervention in a clinical trial, using a control group (aim 3). Guided by community-engaged approaches at all stages of the study, we will collect qualitative, quantitative, and visual data (eg, photographs, community maps, drawings) across multiple districts in Nepal where chhaupadi is practiced.

### Overview of Study Phases and Data Collection Sites

The study will be conducted in several sites across Nepal (Table 1). In aim 1, the RS will be initiated in the capital Kathmandu, where the research team will consult with organizations that have implemented chhaupadi interventions and will collect reports, evaluations, and manuscripts. We will hold a Knowledge Sharing Workshop to which all organizations that have or are currently implementing chhaupadi interventions will be invited to share lessons learned. These organizations will be identified via the Menstrual Health and Hygiene Partner’s Alliance (MHMPA), which tracks the actions of ongoing menstrual health efforts in Nepal [47]. Based on initial findings from an analysis of documentation identified via gray literature and organizations, as well as a review of the literature with the support of a health sciences librarian, follow-up KIIs will be conducted to triangulate findings in several locations across the country. KIIs will be conducted with (1) intervention experts in Kathmandu, (2) staff at the district level where chhaupadi interventions have been implemented, and (3) staff and community members impacted by the interventions in communities where chhaupadi is prevalent. Districts for field-level data collection in aim 1 will be considered if they have a chhaupadi intervention that had/has promising aspects, based on the RS initial analysis.

**Table 1.** Overview of study activities and site types by study aim.

Study activities	Study site types/locations	Inclusion/exclusion criteria
Aim 1: realist synthesis (RS)		
Knowledge sharing workshop (national level) KIIs^a^ with intervention experts (national level) KIIs with implementation staff (district level) KIIs/FGDs^b^ with communities impacted by promising interventions	Kathmandu Districts/communities with promising chhaupadi interventions	Inclusion criteria: Staff of organizations that have implemented chhaupadi interventions Individuals who have received a chhaupadi intervention that had promising aspects, based on RS initial analysis
Aim 2: co-design chhaupadi intervention		
Intervention co-design workshop using IM^c^, HCD^d^, and ABR^e^ methods KIIs - Community dialogue sessions	One chhaupadi practicing district with high prevalence 4 additional medium/high prevalence districts	Inclusion criteria: Chhaupadi-practicing districts with medium or high prevalence of the practice Accessible by jeep within 2 hours from district headquarters Exclusion criteria: Communities with current chhaupadi intervention or within the past 5 years
Aim 3: pilot test		
Clinical trial of community-designed intervention	1 chhaupadi-practicing district 2 matched communities, random allocation to intervention versus control	Inclusion criteria: Chhaupadi-practicing district (medium or high prevalence) Two communities that are sufficiently distant from each other to prevent contamination Exclusion criteria: Communities from previous aims

a KII: key informant interview.

b FGD: focus group discussion.

c IM: intervention mapping.

d HCD: human-centered design.

e ABR: arts-based research.

In aim 2, we will use IM, HCD, and ABR methods to generate unique, contextual, viable solutions with communities to address chhaupadi, based on existing evidence as consolidated through the RS (aim 1) [46,48-53]. In aim 2, a co-design workshop will be held in one “action community” that will be selected based on the latest evidence regarding chhaupadi prevalence. District selection will be informed by the following inclusion criteria: (1) has a high prevalence of chhaupadi, (2) does not currently have an ongoing chhaupadi intervention, and (3) is accessible by jeep (within 2 hours from district headquarters). The exclusion criterion is as follows: (1) communities that have had a chhaupadi intervention in the past 5 years. The SAMIP team will work closely with 10 women in the community, referred hereafter as community design team (CDT) members, to create a chhaupadi intervention by using a series of creative and participatory activities and iterative design tools. Activities for the co-design workshop will be guided by the six steps of IM and will include a series of participatory activities harnessing HCD and ABR tools (Table 2).

**Table 2.** Detailed data collection activities for human-centered design (HCD) co-design workshop (aim 2).

Activity	Description and timing
Intervention mapping (IM) step 1: develop a logic model of the problem^a^	
Develop the logic model of the problem (LMOP) visual (before fieldwork)	Based on a rapid literature review conducted by the SAMIP^b^ team and an RS^c^ study to investigate interventions to date, we will develop a logic model of the health problem. We will also bring in theories that guide the logic model development. This will be translated into a visual figure in Nepali for ease of communication with community members.
Share logic model of the problem visual with community design team (CDT) and gather insights (in the field)	A key visual and clear language in Nepali will be used to communicate key aspects of the LMOP with the CDT. The CDT will be invited to provide feedback on what changes they wish to make by using the HCD tool Rose, Thorn, Bud. Next, the SAMIP team will incorporate the changes into (1) the visual model, and (2) the table model based on CDT feedback. (2 h)
Photo journaling	Photo journaling will be used within an asset mapping exercise to identify key assets in the community to guide the overall study. The CDT will be invited to use iPads in pairs to walk around their community and reflect upon the following prompt: “What are things that you love in or about your community?” The CDT will participate in a group discussion to view and discuss key aspects captured in each photo. (3 h)
Stakeholder mapping	The CDT will brainstorm gatekeepers/key stakeholders of chhaupadi (eg, those who practice, those who perpetuate, and those who have the power to change it). Mindsets for each stakeholder will be discussed. The CDT will vote on 5 key groups of people they deem most important for promoting change. (2 h)
Experience diagramming	The CDT will map their chhaupadi journeys, specifically key aspects of the practice and determine which aspects are liked/beneficial, and which aspects are not liked/could be improved. Participants can use a range of art tools and prepopulated icons. Ideas will be discussed as a group and combined into one combined Experience Diagram based on a group discussion. If needed, the CDT will be provided voting tokens to narrow the scope of the program goals based on (1) major influence on chhaupadi, (2) urgency, and (3) feasibility. (2 h)
Walk-a-mile immersion	The research team will visit CDT households (n=10) to explore how chhaupadi is practiced. This will result in empathy and trust building through first-hand experience and deeper understanding of the range of chhaupadi traditions. (8 h)
Impact ladder	The CDT will explore the lasting social change they wish to see in their community related to chhaupadi. They will also identify immediate outcomes that are expected to contribute to this change. They will specify a target group and timeframe for each goal. (2 h)
Community dialogue session 1 and key informant interviews (KIIs)	We will hold a community dialogue session (n=10) (2 h) and conduct KIIs with key leaders (n=8) (45 min) to share results, gather alternative perspectives, and communicate findings to community members. Findings will also be incorporated into the logic model of the problem to ensure a holistic understanding of the intervention context, including the setting and community and assets. Discussions will be audio-recorded.
IM step 2: specify program outcomes (ie, target behaviors), performance objectives, and logic model of change^d^	
Review impact ladder and share community dialogue session insights with CDT	The research team will summarize the insights gathered from the community dialogue session. The CDT will have an opportunity to revisit their impact ladder, refine, and edit based on community input. (1 h)
Change objectives, performance objectives, and logic model of change (internal preparation with CDT feedback)	Based on the insights gathered using the impact ladder review, the research team will compile the findings into a matrix of change objectives tool used in IM. The research team will share the draft matrix with the CDT members using icons and visuals. After incorporating CDT feedback, the research team will compile the feedback and draft a Logic Model of Change using the templates from the IM process. (3 h)
IM step 3: program design^e^	
Importance difficulty (ID) matrix	As a group, the CDT will decide where change objectives fall on an ID Matrix. On a large piece of paper, there will be an option of 4 quadrants (low difficulty, low importance; low difficulty, high importance; high difficulty, high importance; and high difficulty, low importance). Using icons for each program objective, CDT members will place them in the appropriate quadrant depending on how difficult it would be to implement, and the importance for bringing about change related to chhaupadi. (2 h)
Buy a feature	Each quadrant of the ID will be assigned a price. Participants will be given currency notes, and they will be asked to “purchase” program components within a limited budget. This will allow the CDT to explore the importance of specific activity combinations and provide an opportunity for the CDT to consider how different program components might fit together in an intervention. (2 h)
Methods for changing behavior presentation	The SAMIP team will provide an overview of evidence-based approaches to changing behavior, which participants can draw from for future exercises. (30 min)
Concept posters	The CDT will break into teams of 2 people to develop Concept Posters for the program components that they “purchased” in the previous “Buy A Feature” exercise. Each team of 2 people will be provided a poster with key areas to consider for their intervention development and will be invited to use creative supplies (eg, paints, icons, drawing and writing utencils, etc). Posters will include the following prompts: intervention goals, desired outcomes, target groups, key implementers, main components, locations, duration, and inputs required. Upon completion, they will pitch their ideas to the wider CDT. (4 h)
Rose, thorn, bud	After pitching concept posters, the CDT will use the rose, thorn, bud technique to identify aspects of the intervention that are expected to be successful (“roses”), aspects that may fail (“thorns”), and aspects that have potential but could be improved (“buds”). (2 h)
Visualize the vote	The CDT will vote for the 5 intervention components they like the most, particularly those that they believe will bring about the greatest change. (2 h)
“Coolers” color palette brainstorm	The CDT will be invited to brainstorm what colors come to mind based on their ideal chhaupadi program that they have designed in the previous steps. Everyone picks a color and explains why they selected it. (1 h)
Dream and design	The CDT will be asked to go around the community and gather materials (ie, natural elements like leaves, sticks, rocks, dirt, spices, makeup, cloth, etc) that could be used to communicate or represent their chhaupadi program. They will come back together to create a rangoli-like collage, present, and discuss. Throughout the discussion, the research team will seek to identify common themes, symbols, words, etc, around their ideal chhaupadi program. The CDT will also finalize the title of the intervention. These created materials will be used in the intervention materials to ensure it is grounded in the vision, designs, and creative ideas of participants. (4 h)
Model making	The CDT will break into teams to create “sample” activities and outputs related to their intervention. For example, one group may create a sample video if they wish to use social media videos and campaigns in their intervention. The CDT will share their outputs and creations with the full CDT. (3 h)
Visual voices	As a closing visioning exercise, the CDT will participate in visual voices. They will complete a blind drawing, and painting and writing exercises to ultimately create a group collage/mural reflecting on the following prompt: If your intervention is successful, what will your community look and feel like? (4 h)
Community validation meeting 2	The same community members from the community validation meeting 1 will be invited back to review the CDT concept posters, provide feedback, or offer alternative insights. They will vote on the intervention ideas that they believe will have the greatest impact. (2 h)
Focus group discussion (FGD)	The CDT will participate in an FGD, with the goal of understanding experiences using HCD to develop interventions, explore how the activities impacted them, what worked well, what could be improved, and ideas for the future. (1.5 h)

a Goal: Finalize a logic model of the problem (LMOP), which incorporates findings from realist synthesis (RS; aim 1), with input from the community.

b SAMIP: Strengthening Actions for Menstrual Health and Hygiene Interventions for Promotion of Women’s Health in Nepal.

c RS: realist synthesis.

d Goal: Specify what changes related to chhaupadi and the surrounding environment need to occur to improve health and quality of life.

e Goal: Design a chhaupadi intervention that is evidence-based and incorporates creative ideas generated from the CDT using HCD and ABR tools, with input from community members.

After initial intervention ideas are developed in partnership with the action community, we will conduct a “rapid situation analysis,” where local data enumerators will elicit feedback on the initial intervention ideas in an additional 4 chhaupadi-practicing districts with medium or high prevalence of the practice, known as “collaboration communities.” Enumerators will summarize key findings from the HCD workshop, share the intervention concept with a variety of community members in the collaboration communities, and collect feedback on perceived strengths, areas for refinement, and contextual barriers to implementation. Insights gathered from collaboration communities will be critical to generating insights to guide contextual adaptation of the intervention across districts and to equip the team for effective scale-up of the approach. Finally, the team will hold an “insight workshop” at the community level to share insights gathered from the rapid situation analysis and share a final intervention concept via a one-day workshop.

In aim 3, we will conduct a clinical trial to pilot test the intervention approach in one chhaupadi-practicing community and include a control group. One chhaupadi-practicing district will be selected, but it will exclude communities visited during the previousaims. The control and pilot communities will be matched and will be far enough away to prevent spillover effects.

### Study Population and Recruitment

For all aims, individuals will be included across the lifespan, age 15 years and older; there is no maximum age range for the study overall. Given the focus of this study on practices (ie, chhaupadi) that specifically impact women and girls who currently experience monthly menstruation, or have experienced menstruation, the minimum age for enrollment is 15 years. The study will be open to men, women, and gender-expansive individuals, as all these groups are expected to play key roles in the menstrual movement in Nepal and, therefore, will be eligible to participate in various aspects of the research study if they meet the inclusion criteria for each aim.

For aim 1, key informant participants at the national level must be current or previous members of a team/organization that has implemented a chhaupadi intervention in Nepal, and have expertise in chhaupadi interventions (eg, a representative from the Family Welfare Division). For the district and community level KIIs with program staff, participants must currently work or have worked at the time of the intervention for an organization implementing a chhaupadi intervention that is included in the RS. Community-level interviews will also be conducted with those who were direct recipients of the chhaupadi intervention, or who are leaders in the community where the chhaupadi intervention took place, and can speak to the effectiveness and response to the intervention. Those under the age of 15 years will be excluded. We aim to enroll a minimum of 35 participants in aim 1.

For aim 2, we will establish one CDT of 10 participants to work with the study team to co-design a chhaupadi intervention. Sample size is guided by standards in the literature for the ideal focus group size. The CDT will be comprised of women and girls who practice chhaupadi (menstruating age and older), from diverse backgrounds including occupation, age, caste/ethnicity, religion, literacy, and marital status. CDT participants will be recruited via snowball sampling and through community-level collaborators who have strong ties with local NGOs and government leaders. The CDT and all participants will be compensated for their time. Community dialogue sessions (2, n=10) will include youth, elders, religious leaders, teachers, health care workers, and other community members who can speak to the potential for a chhaupadi intervention in the community. We will also conduct KIIs (n=8) with key community leaders such as local police, government leaders, and activists with chhaupadi expertise to inform our overall understanding of the context.

The “rapid situation analysis,” will focus on gathering quick snapshots from a variety of chhaupadi-practicing districts, from a range of community members, including key informants (eg, police, teachers, health post in charge, members of government), to those with lived experience practicing chhaupadi. The participants will be selected in partnership with the local municipality office of each study district.

Finally, the “insight workshop,” which will be held at the district level in one of the chhaupadi-practicing districts to ensure accessibility for community members and local leaders, will bring together the original CDT members from aim 2.

In aim 3, two communities (1 intervention and 1 control) will be selected in one chhaupadi-practicing district of Nepal with high prevalence of chhaupadi based on the latest evidence at the time of data collection. We seek to identify communities that have not been exposed to a chhaupadi intervention in the past 5 years. Control and intervention communities will be matched based on exposure to chhaupadi interventions, population, rurality, and caste/ethnic makeup.

Eligibility criteria for the clinical trial will be refined based on the target groups of the final intervention developed in aim 2, but are expected to include women and girls who currently practice chhaupadi and have been residents of the communities for at least one year. We will use a sample size that allows for preliminary testing of feasibility and acceptability. Before enrolling, the research team will screen participants based on eligibility criteria, obtain informed consent, and document reasons for nonparticipation, if necessary. Communities will be randomized into the intervention or control group to eliminate bias; the control group will receive health education materials only.

### Intervention Material Development

After aim 2 activities are complete, we will hire a Nepal-based educational consultant to review plans and materials, and to support with drafting the intervention materials that meet the vision of the CDT members. The consultant will also partner with the SAMIP team to develop an implementation plan, which will consider timing, locations, key players, and other implementation logistics. Additionally, we will partner with a graphic design company to ensure the materials are created, designed, and executed according to the vision of the CDT members. The graphic design team will be responsible for the design and finalizing the layout, look, and feel of the intervention materials, which will include written content (in English, Nepali, and local dialect, if necessary), illustrative graphics, an implementation guide, intervention sequence, training instructions, and other materials as needed. Overall, both the educational consultant and the graphic design company will help finalize intervention materials in preparation for the clinical trial in aim 3.

### Data Collection Activities

#### Aim 1: RS

We seek to extend the literature by assessing existing chhaupadi interventions by exploring what works, for whom, and under what circumstances (ie, mechanisms of success). To do so, we will build upon extant studies by (1) conducting an RS to classify complex, heterogeneous intervention data; (2) triangulating intervention data with KIIs at community, district, and national levels; and (3) assessing underlying theories of interventions.

In Step 1, we will define and clarify the scope of the review, identify initial intervention theories, and conduct a preliminary search. This process will include 3 brainstorming sessions with menstrual health program experts from the MHMPA to (1) refine the research question; (2) define key terms relevant to the search (eg, How is chhaupadi defined? What are the parameters of a chhaupadi intervention?); (3) finalize search strategies; (4) develop a draft theoretical framework; (5) set study rigor threshold; and (6) finalize data extraction fields.

In Step 2, we will search and appraise the evidence. The research team will develop a search strategy and timeline, then 2 reviewers will screen titles and abstracts against inclusion/exclusion criteria using bespoke extraction forms in DistillerSR; the form will be pretested with a subset of data until disagreements are resolved. Studies/evaluations will be included if they (1) address chhaupadi via an intervention in Nepal (eg, behavioral, educational, policy, media, health systems, etc), or (2) address chhaupadi as a component of a broader intervention (eg, chhaupadi educational material as a part of a broader sexuality education program). Studies/evaluations will be excluded if they (1) are anecdotal, (2) are not relevant to chhaupadi, (3) are not based in Nepal, or (4) are published in a language other than English or Nepali.

In Step 3, the team will extract and synthesize evidence. The extraction tool will include questions related to the theory of change and the central review question and will include direct quotes from data sources. Given the iterative nature of RS, we will add additional information that is deemed important for informing our theoretical framework throughout the process, as is standard practice [54]. We will assess each study/intervention for rigor and quality by using established ranking criteria [55], and credibility and trustworthiness criteria [44]. Only studies that meet the agreed-upon threshold will be included; notably, even lesser quality evidence can be relevant for theory development in RS [56].

To reduce biases and increase the validity of the data collected via secondary sources, we will conduct interviews at the national (n=10), district (n=10), and community levels (n=15) to verify findings with experts who have implemented chhaupadi interventions, similar to Kantilal et al [55], approach for capturing real-world feedback. Sample size was determined based on recommendations from empirical studies on qualitative saturation, in which saturation is often reached within 9 interviews [57]; however, additional interviews will be added if saturation is not reached (Multimedia Appendix 1 provides details on topic guides).

In step 4, presentation of our findings will be organized according to the theoretical framework developed and refined throughout the RS, using constructs of context, mechanism, and outcome. Recommendations will be established via a final brainstorming session with experts from Step 1. Results will be reported according to the Realist and Metanarrative Evidence Synthesis Standards for quality and publication (RAMESES; Multimedia Appendix 2 provides details on the checklist) [44].

#### Aim 2: Intervention Cocreation

Guided by findings from aim 1, in aim 2, we aim to co-design a chhaupadi intervention that meets the unique contextual needs of communities while also incorporating current evidence and theory. This study will significantly build upon the intervention development literature by harnessing the systematic and theoretical approaches offered by IM, combined with the creative and community-centered benefits that HCD and ABR methods offer.

Drawing inspiration from Leung et al’s HCD model [58] and refined through pilot testing [41,48], we will use the six steps of IM [46] combined with several creative and participatory activities to develop a contextual understanding of gatekeepers of chhaupadi, their mindsets, experiences of chhaupadi, and drivers and facilitators of the practice. We will work with an action community to co-design a chhaupadi intervention, along with community dialogue sessions and conduct KIIs to share results, gather alternative perspectives, and communicate findings to community members (Table 2).

The design and implementation of the co-design workshop will be overseen by a PhD-trained researcher with expertise in participatory and community-engaged methods, who identifies as a woman, a mother, and a menstruator, which is expected to support in establishing rapport and trust with participants. The PI has intermediate Nepali language skills and will be supported by additional research assistants who are fluent in English and Nepali, including the local dialects, to ensure all activities are understandable for all participants and team members.

Next, local data enumerators will conduct a rapid situation analysis (n=75), in which they will summarize key findings from the HCD co-design workshop and share with community members and leaders in several chhaupadi-practicing districts. Through KIIs, they will gather feedback and considerations for contextualizing the intervention and future scale-up. Finally, the CDT will be invited to give feedback during an insight workshop, in which we will conduct several creative and participatory activities to gather insights to finalize the intervention design and implementation plan (additional data collection activities outlined in Table 2).

#### Aim 3: Pilot Trial

We will pilot test the chhaupadi intervention in one community and include a matched control community located sufficiently far away to minimize spillover effects. The trial will focus on assessing feasibility and acceptability, as well as collecting preliminary data on knowledge, attitudes, behaviors, and health outcomes expected to be impacted by the intervention. The intervention will be delivered by trained community organizers with expertise and experience conducting health interventions in rural Nepal. As a research organization, our partner Bikash Shrot Kendra will provide support with intervention evaluation and ensure implementation is consistent across pilot communities. We estimate that the intervention will include 8‐10 two-hour sessions in the community, over the course of 4 months, estimated based on other intervention approaches for shifting cultural norms.

Quantitative outcome measures will be used to assess feasibility and acceptability, and results will inform the design of a subsequent trial. We will conduct focus group discussions (FGDs) with participants and the intervention team to discuss challenges, barriers, and opportunities to adjust the intervention material and protocol (mid-point and post). Last, we will follow up with participants six months after completion to further assess outcomes.

### Analysis

#### Overview

We will use StataSE for quantitative analyses. For qualitative analyses, all audio-recorded data will be transcribed and translated from Nepali to English by trained translators who are fluent in both languages. Photographs, visuals, and diagrams collected as part of the HCD activities will also be transcribed/translated using standardized spreadsheets in Microsoft Excel. Qualitative analyses will be conducted in Dedoose. For all aims, we will collect demographic information about each study participant, which will be used to describe the study sample based on key demographic variables. Intervention reporting will follow the Template for Intervention Description and Replication (TIDieR) checklist (Checklist 1 provides more information).

#### Specific Analysis Plans for Aim 1

In the analysis of studies/intervention reports for the RS, we will scrutinize the evidence, specifically each study/intervention by context, mechanisms, and outcomes, aligned with RS standards [45]. Context will explore the conditions under which the intervention impacts outcomes (eg, geographic location and cultural norms). Mechanisms, or processes operating within an intervention, will explore how participants use the resources offered through the intervention. Outcomes will include the consequences of the study/intervention, intended or unintended, resulting from the interaction between context and mechanism (eg, improved mental health outcomes and reduced number of women practicing chhaupadi) [56]. We will use these components to describe the extent and circumstances under which an intervention works or does not, and to test and refine our theoretical framework drafted in Step 1. Screened data will be exported from Qualtrics forms and discussed with the review team to create data evidence tables [45] organized by key theme (eg, context, mechanism, and outcome). Next, we will assess the data for a chain of inference to explore connections across studies/interventions, as is standard practice in RS [45,56].

KIIs (conducted in Nepali or English based on participant preference) will be audio recorded, transcribed, and translated into English as necessary. Two coders will analyze data thematically in NVivo (Lumivero). Presentation of our findings will be organized according to the theoretical framework developed and refined throughout the RS, using constructs of context, mechanism, and outcome. Recommendations will be established via a final brainstorming session with experts from Step 1. Results will be reported according to the RAMESES for quality and publication [44].

#### Specific Analysis Plans for Aim 2

The resulting data from the HCD workshops will include the following. (1) Field notes: reflections on what worked effectively and what could be improved. (2) HCD artifacts: audio recordings and photographs of the outputs for all HCD activities (eg, stakeholder map, walk a mile immersion, photo journaling, experience diagram, impact ladder, etc) transcribed/translated into standardized Excel sheets for further analysis. This will include direct quotes from the activity discussion from audio recordings, which will be transcribed and translated into English. (3) FGDs and KIIs: audio recordings of FGDs and KIIs will be transcribed and translated from Nepali to English. All data will be qualitatively analyzed thematically in Dedoose and guided by the Health Stigma and Discrimination Framework [59].

Based on findings from the co-analysis workshops, the PI will lead the development of the intervention materials, implementation protocol, and training manual. The educational consultant will be responsible for creating presentations providing overviews of each intervention module, including material lists, goals, and activity instructions. Intervention materials will include written content (in English, Nepali, and local dialect, if necessary), illustrative graphics, an implementation guide, and activity instructions. The graphic design company will ensure that all intervention materials, such as training instructions or an implementation guide, are in accordance with the vision of the CDT members. Based on piloting, we posit that the core intervention could include (1) activities at multiple levels of the social ecological model, considering the influence of individual knowledge, family beliefs, social pressure, and policies on menstrual behaviors; (2) multiple target groups for behavior change, including but not limited to mothers/women, youth, female community health volunteers, and religious leaders, each with their own behavior change messaging and approaches (eg, community group sessions vs radio/newspaper vs one-on-one education); and (3) sessions over the course of several months, since social norms and behaviors require considerable time to shift.

We will elicit feedback on the intervention content and protocol using cognitive interviewing, an evidence-based method for determining if the proposed tool fulfills its planned purpose [60], with 10 experts from MHMPA and BSK before the pilot test in aim 3.

#### Specific Analysis Plans for Aim 3

This pilot is not intended to power between-arm comparisons; however, findings will allow for an initial assessment of feasibility and acceptability in preparation for a future randomized controlled trial. Aligned with pilot study best practices, we will describe the intervention sample using participant characteristics (n and % for categorical variables and mean and SD for continuous variables). We will calculate the approached-to-enroll and completion rates compared with prespecified benchmarks (Table 3) and report the percent of sessions completed as planned, participant completion rates, as well as outcomes for each measure (frequency and %, mean and SD, or median and range). Since this is an initial pilot test, no formal efficacy tests will be conducted by the comparison group; however, we will run exploratory analyses on outcomes by caste/ethnicity, age, education, and religion for descriptive purposes only. We will also test for differences in potential confounders between the intervention and control groups and adjust for them in regression models. All quantitative analyses will be conducted in Stata SE (StataCorp LLC).

**Table 3.** Measures and tools for aim 3 data collection.

Measures/tools	Pre	Mid	Post	6 month
Demographics (eg, sex, caste/ethnicity, religion, education, occupation, and age)	✓			
Intervention Pilot Testing Measures				
Enrollment: Enrollment pace, and retention (Benchmark: ≥80% of approached individuals enrolled; ≥80% retention)			✓	
Acceptability: For participants and implementers, acceptability of intervention measure [61] a valid and reliable measure of acceptability with 4 items on a 5-point Likert (Benchmark: ≥3.5) and open-ended questions on acceptability of intervention components			✓	
Appropriateness: For participants, the intervention appropriateness measure [61] a valid and reliable measure of appropriateness with 4 items on a 5-point Likert scale (Benchmark: ≥3.5)			✓	
Feasibility: Extent to which the intervention is successfully executed. Feasibility of Intervention Measure [61] a valid and reliable measure of feasibility with 4 items on a 5-point Likert scale (Benchmark: ≥3.5)			✓	
Challenges, barriers, opportunities: FGDs^a^ with implementers (n=1) and participants (n=2)		✓	✓	
Exploratory intervention outcome measures				
Mental Health: Beck Anxiety Inventory Validated for Nepal [62] 21 items, 4-point Likert	✓		✓	✓
Reproductive Health Questionnaire [17]	✓		✓	✓
Chhaupadi knowledge, attitudes, and behaviors	✓		✓	✓
Self-efficacy: Generalized Self-Efficacy Scale, 10 items, 4-point Likert [63]	✓		✓	✓

a FGD: focus group discussion.

Qualitative analyses of open-ended and FGD data will be coded thematically in NVivo. We will professionally transcribe and translate the data, and 2 independent members of the research team will analyze data using template analysis to enhance rigor, allowing for inductive and deductive analysis [64].

### Ethical Considerations

Given the stigmatized nature of menstruation in Nepal, as well as the legal measures banning and criminalizing the practice, it is necessary to take every precaution to prevent and mitigate risk or harm to participants. To that end, this study received approval from both the University of Pittsburgh Institutional Review Board (study number: MOD23080043-001) as well as the Nepal Health Research Council Ethical Review Board (Protocol Number: 640_2023). Every effort will be made to ensure there are no changes in the risk/benefit ratio during the study and that confidentiality of research data is maintained. All records related to this study will be stored in secure databases only accessible by research staff or in a locked file cabinet. Additionally, all research staff have completed training on the ethical principles of research, highlighting confidentiality to minimize the risk of breaches of confidentiality.

The research team will obtain informed consent from every adult participant in a manner that is suitable to the context. In aim one, the research team will collect consent via a Qualtrics survey via mobile device or pen and paper, given the participant’s preference and technological literacy. In aim 2, informed consent for participation in the co-design workshops, community validation meetings, KIIs, and cognitive interviews will be obtained in person via Qualtrics on a mobile device prior to collecting data. Obtaining informed consent for pilot testing the intervention in aim 3 will follow similar procedures to aim 2.

Participants younger than 18 years will be asked to provide assent, and the research team will ask a parent or guardian to sign the consent form. All consent forms will outline the following details: goals of the study; foreseeable risks or potential discomfort; how confidentiality will be maintained; potential benefits; contact information for the PI and research assistant; information about the voluntary nature of the research study, that they can withdraw at any time, and can skip any questions that they do not wish to answer. Consent materials will be available in English and Nepali, and participants can choose which language they prefer.

To minimize coercion and undue influence, participants will be informed about the study in advance of the study team coming to the village, so they will have ample time to reflect on whether they would like to participate. We will also hold an informational meeting with potential participants to answer any questions that they may have before initiating the consent process.

The research team developed additional safety plans for the pilot study in aim 3 to address unlikely but possible inadvertent effects of the intervention on stigmatizing the practice. This includes (1) continuously monitoring community impacts of the intervention design and associated discussions (eg, through community validation meetings and KIIs); (2) stigma will not be used to promote global health in any aspect of the chhaupadi intervention [65], which will be discussed with the CDT during aim 2; (3) applying an intersectional lens throughout the study to ensure that intersecting forms of one’s identity are considered and protected throughout the study (eg, caste/ethnicity, religious, gender, rurality, marital status, education); (4) establishing a community safety advisory board (CSAB) in the intervention community to focus on risk management. During the intervention implementation, the PI will meet with the CSAB monthly to monitor data collection, management processes, examine the integrity of data analysis, and review feedback. Any concerns regarding potential risk to the participants and broader community will be seriously considered, and the PI will develop rapid risk mitigation plans as needed; (5) implementing measures in pre, mid, and postintervention surveys to assess shifts in stigma in real-time within the intervention community. This will allow the team to capture potentially negative impacts early and address them in partnership with the CSAB; and (6) asking directly about stigma experiences in FGDs at the mid and end points of the intervention to better understand the nature of stigma experiences and how they may be impacted by the intervention.

## Results

The study was funded in September 2023. Aim 1 activities began in April 2024. During the review, the study team identified 2033 resources for potential inclusion in the RS. After reviewing, 69 materials met the inclusion criteria and were analyzed in detail, which included peer-reviewed manuscripts, evaluation documents, program reports, videos, and more. To supplement findings from secondary sources, a workshop with 8 organizations was held in the capital to generate initial chhaupadi program theories and identify initial drafts of causal mechanisms for further analysis. These initial program theories and insights from the secondary sources were discussed at the national and district levels via consultations with 44 individuals. Analysis was completed in February 2026. Evidence suggests that sustainable change requires the creation of enabling environments, incremental behavior change, and the active involvement of families and local leaders, alongside attention to structural determinants such as economic insecurity and women’s financial empowerment. Full results are expected in Summer 2026.

Aim 2 data collection began in March 2025. A 10-day co-design workshop using HCD tools and ABR methods was implemented in Dailekh with 11 women and CDT members, and they created a draft intervention titled, हाम्रो आवाज: छाउपडी अन्त्य परिवर्तित जीवन (Our Voice: Transforming Lives through Ending Chhaupadi). An additional 123 community members have been consulted, both in the design community as well as across 4 additional districts (Achham, Baitadi, Doti, and Surkhet). The refinement of the intervention is ongoing based on community feedback. Results are anticipated in Fall 2026.

Aim 3 data collection is projected to begin in Winter 2027.

## Discussion

### Principal Findings

This study protocol offers several methodological innovations for global health intervention synthesis and development. It is the first known study to harness IM, partnered with HCD and ABR methods for intervention development in Nepal. The approach outlined in this paper builds upon pilot work our team conducted in Dailekh, Nepal, in 2022 [41,48,50].

This study protocol offers several methodological innovations to the field of global women’s health. We are the first to apply RS to menstrual health in Nepal. Unlike previous approaches, before developing an intervention, we will first assess interventions, enabling us to understand what elements work, in what contexts, and how [45].

This is also the first known example of partnering with communities to co-design an intervention on this topic and with this population. Although several interventions have targeted chhaupadi, such as elimination campaigns, community sensitization, and radio shows, the practice persists in parts of the country [38]. Other initiatives have dismantled menstrual sheds or withheld state support services, but have often resulted in increased vulnerabilities, forcing women to live in harsher conditions [37,66]. Shortcomings of these interventions (eg, top-down approaches) have prevented advancement of the field, likely because they lack the integration of community voices in all aspects of the intervention design, implementation, and assessment. Our preliminary pilot evidence suggests a community-engaged approach will result in a locally relevant response to shifting deep-rooted behaviors. This protocol offers HCD and ABR tools as relevant ways in which communities with diverse abilities, expertise, and backgrounds can be incorporated at all stages of the intervention design and planning process.

Finally, this will be a novel use of HCD and ABR methods for intervention development in Nepal. While HCD has been successfully applied to co-design interventions in several contexts in which culture and social norms play key roles [58,67], including for reproductive health [68,69], this will be the first application of HCD to develop a full menstrual health intervention in Nepal. By developing and piloting the intervention, guided by IM principles, we will contribute significant knowledge of culturally appropriate intervention attributes that can be scaled and rigorously tested in future studies.

### Limitations

While our study is built on the strengths of community engagement and theory- and evidence-based intervention development, it is not without limitations. A key limitation of the RS in aim 1 is that its findings depend on the quality and detail of existing literature and stakeholder input, which may vary and result in incomplete or context-specific explanations that are not universally generalizable. While we will seek to recruit a diverse sample throughout all aims, we are limited by time, budget, and transportation/road access, and therefore, our sample is not representative of all communities and individuals who partake in the chhaupadi tradition. Additionally, while community members will design intervention concepts, provide multiple rounds of feedback, and we will triangulate the findings in multiple districts, we anticipate that additional intervention development design work will be required by the research team to turn the community ideas into replicable intervention activities. Additional contributions from the research team, designers, content area experts, trainers, and program developers will be harnessed to build upon the foundation established by community members. We will incorporate the voices of the community advisory board as we develop the protocols, training manuals, and tools, but this part of the process will primarily be led by the research team and is a limitation due to time and budget. Finally, limitations in our timeline and budget may influence the complexity of the intervention that we are able to fully develop and test.

### Conclusions

Despite numerous efforts, a comprehensive understanding of the effects of chhaupadi interventions across Nepal remains absent. This study will use RS to identify what works, for whom, and under which circumstances. Insights from the RS will directly inform co-designed interventions with community members, leveraging IM, HCD, and ABR methods. By embedding diverse community perspectives at every stage of the study, this approach ensures interventions are contextually grounded and culturally relevant. Beyond addressing a culturally situated menstrual health practice—chhaupadi—this community-centered model has the potential to inform strategies for reducing the harms associated with other harmful practices in diverse global settings.

## Supplementary material

10.2196/89117 Multimedia Appendix 1 Interview and focus group topic guides.

10.2196/89117 Multimedia Appendix 2 RAMESES publication standards for realist synthesis.

10.2196/89117 Checklist 1 The TIDieR checklist.

10.2196/89117 Peer Review Report 1 Peer review report by the ZRG1 ICP-P (55) - Center for Scientific Review Special Emphasis Panel, PAR Panel: International and Cooperative Projects (National Institutes of Health, USA).

## Abbreviations

ABR: Arts-Based Research
CDT: Community Design Team
CSAB: Community Safety Advisory Board
FGD: Focus Group Discussion
HCD: Human-Centered Design
IM: Intervention Mapping
KII: Key Informant Interview
MHMPA: Menstrual Health and Hygiene Partner’s Alliance
RAMSES: Realist and Metanarrative Evidence Synthesis Standards for quality and publication
RS: Realist Synthesis
SAMIP: Strengthening Actions for Menstrual Health and Hygiene Interventions for Promotion of Women’s Health in Nepal
TIDieR: Template for Intervention Description and Replication
