# Supplementary material for: Strengthening Actions for Menstrual Health and Hygiene Interventions for Promotion of Women’s Health in Nepal (SAMIP): Protocol for a Participatory Intervention Development Study Using Realist Synthesis, Human-Centered Design, Intervention Mapping, and Arts-Based Methods
Source: JMIR Res Protoc. 2026 Apr 23;15:e89117. doi: 10.2196/89117 (PMC13105233; doi:10.2196/89117)
Supplement: Multimedia Appendix 1 [file resprot-v15-e89117-s001.docx]

**Appendix 1. Interview and Focus Group Topic Guides**

The following interview and focus group guides represent draft, semi-structured instruments developed to align with the study aims. Given the multi-phase and iterative nature of this research, several data collection tools—particularly for Aims 2 and 3—will be refined based on findings from earlier phases (e.g., Realist Synthesis results and co-design workshop outputs). The guides are therefore intended to outline core domains of inquiry while allowing flexibility to probe emergent themes, contextual dynamics, and evolving program theories. This adaptive approach is consistent with realist and community-engaged methodologies, which prioritize responsiveness to context and ongoing theory refinement.

## Aim 1: Realist Synthesis

### Interview Topic Guide – Organizations and Experts Working to Address Chhaupadi in Nepal

### A. National-Level Stakeholders

**1. Overview of Organizational Work on Chhaupadi**

- Please describe your organization’s work related to chhaupadi.
  - Name and duration of the intervention/program
  - Key components and activities
  - Theories, frameworks, or guiding principles informing the intervention
  - Target populations
  - Geographic focus (districts/regions; rural/urban context)
  - Implementation timeline
  - Monitoring and evaluation approaches
  - Key findings or outcomes (if available)
- Reflections on implementation:
  - What have been the main facilitators of success?
  - What barriers or challenges have you encountered?

**2. Mechanisms of Change**

- In your view, how and why did (or did not) your intervention influence chhaupadi-related practices?
- What underlying social, cultural, or structural mechanisms were most important?

**3. Outcomes and Impact**

- What were the primary intended outcomes of your program?
- Were there any unintended outcomes (positive or negative)?

**4. Sustainability and Broader Context**

- In your opinion, why has chhaupadi persisted despite ongoing efforts?
- What factors make elimination or reduction particularly challenging?

### B. Field-Level Implementers

**1. Overview of Organizational Work on Chhaupadi**

- Please describe your organization’s activities related to chhaupadi in this district.
  - Key components and activities
  - Target groups
  - Implementation context (community setting, timing, local dynamics)
  - Observed results or changes
  - Barriers and facilitators
- Are there other organizations working on chhaupadi in this area? If so, how do efforts align or differ?

**2. Current Chhaupadi Context**

- How is chhaupadi currently practiced in this community?
  - Prevailing norms and beliefs
  - Changes over time
  - Perceived health, safety, or social impacts

**3. Refinement of Initial Program Theories**
(After presentation of preliminary context–mechanism–outcome configurations)

- What aspects resonate with your experience? Why?
- What do you disagree with? Why?
- What is missing or needs refinement?

## Aim 2: Co-Design of Chhaupadi Intervention

### Interview Topic Guide – Community Members, Key Influencers, and Community Leaders

**1. Community Context**

- Please describe how chhaupadi is practiced in your community.
  - Specific practices and expectations
  - Perceived impacts (health, safety, social, economic)
  - Reasons people continue to follow the practice
  - Personal perspectives

**2. Past and Current Efforts**

- What efforts have been made in this community to address harms associated with chhaupadi?
  - What worked well?
  - What challenges were encountered?
  - Are these efforts ongoing? Why or why not?

**3. Future Solutions and Intervention Design**

- What do you believe would be effective ways to reduce harms associated with chhaupadi in your community?
  - Suggested activities or strategies
  - Who should be involved or targeted?
  - Appropriate duration and intensity
  - How should the community be engaged?

## Aim 3: Pilot Intervention

### Focus Group Discussion Guide – Intervention Participants and Implementers

**1. Overall Experience**

- What aspects of the intervention are working well?
- What changes have you observed (if any)?

**2. Implementation Challenges**

- What challenges have arisen during implementation?
- What barriers have participants or implementers faced?

**3. Acceptability and Feasibility**

- How acceptable is the intervention to participants and the broader community?
- Is the intervention feasible to continue in this setting? Why or why not?

**4. Recommendations for Improvement**

- What should be improved, added, or removed?
- Are there elements that need immediate adjustment?
- What would strengthen sustainability?
