# Supplementary material for: Strengthening Actions for Menstrual Health and Hygiene Interventions for Promotion of Women’s Health in Nepal (SAMIP): Protocol for a Participatory Intervention Development Study Using Realist Synthesis, Human-Centered Design, Intervention Mapping, and Arts-Based Methods
Source: JMIR Res Protoc. 2026 Apr 23;15:e89117. doi: 10.2196/89117 (PMC13105233; doi:10.2196/89117)
Supplement: Multimedia Appendix 2 [file resprot-v15-e89117-s002.docx]

**Appendix B. RAMESES Publication Standards for Realist Syntheses**

List of items to be included when reporting a realist synthesis

| **Section** | **Item No.** | **Checklist Item** | **Reported (Y/N)** |
| --- | --- | --- | --- |
| **Title** | 1 | Identify the document as a realist synthesis or review | Y |
| **Abstract** | 2 | Include brief details of: background, review question/objectives, search strategy, methods of selection/appraisal/analysis, main results, and implications for practice | Y |
| **Introduction** | 3 | Rationale for review: Explain why the review is needed and its contribution to existing understanding | Y |
|  | 4 | Objectives and focus of review: State objectives/review questions and rationale for focus | Y |
| **Methods** | 5 | Changes in the review process: Describe and justify any deviations from the planned process | Y |
|  | 6 | Rationale for using realist synthesis: Explain why this method was selected | Y |
|  | 7 | Scoping the literature: Describe and justify the initial exploratory scoping | Y |
|  | 8 | Searching processes: Describe iterative search strategy, sources, databases, search terms, coverage dates, and expert contacts | Y |
|  | 9 | Selection and appraisal of documents: Explain inclusion/exclusion criteria and justification | Y |
|  | 10 | Data extraction: Describe which data were extracted and justification | Y |
|  | 11 | Analysis and synthesis processes: Detail constructs analyzed and analytic/synthesis procedures | Y |
| **Results** | 12 | Document flow diagram: Provide number of documents assessed, included, excluded, and reasons, indicating sources | Y (Full details in the RS manuscript but described briefly in results section of protocol paper) |
|  | 13 | Document characteristics: Describe included documents | Y |
|  | 14 | Main findings: Present key findings focusing on theory building and testing | Y (preliminary) |
| **Discussion** | 15 | Summary of findings: Summarize main findings in relation to objectives/questions and intended audience | N/A (Will include in Aim 1 manuscript, N/A in the protocol paper as results are not fully available yet) |
|  | 16 | Strengths, limitations, and future research directions: Discuss review strengths, limitations, and areas for future work | Y |
|  | 17 | Comparison with existing literature: Compare/contrast findings with other relevant literature | N/A (Will include in Aim 1 manuscript, N/A in the protocol paper as results are not fully available yet) |
|  | 18 | Conclusion and recommendations: State main implications, including policy/practice recommendations where appropriate | N/A (Will include in Aim 1 manuscript, N/A in the protocol paper as results are not fully available yet) |
|  | 19 | Funding: Provide source of funding, funder role, and conflicts of interest | Y |
