# Supplementary material for: Strengthening Actions for Menstrual Health and Hygiene Interventions for Promotion of Women’s Health in Nepal (SAMIP): Protocol for a Participatory Intervention Development Study Using Realist Synthesis, Human-Centered Design, Intervention Mapping, and Arts-Based Methods
Source: JMIR Res Protoc. 2026 Apr 23;15:e89117. doi: 10.2196/89117 (PMC13105233; doi:10.2196/89117)
Supplement: Peer Review Report 1 [file resprot-v15-e89117-s004.pdf]

SUMMARY STATEMENT
(Privileged Communication)

PROGRAM CONTACT:
[redacted]

Release Date: [redacted]
Revised Date:

Application Number: [redacted]
Formerly: [redacted]

Principal Investigator

[redacted]

Applicant Organization: [redacted]

Review Group: [redacted]
Center for Scientific Review Special Emphasis Panel
PAR Panel: International and Cooperative Projects

Meeting Date: [redacted]
Council:
Requested Start:

Opportunity Number: [redacted]
PCC:
Dual PCC:
Dual IC(s):

Project Title: **Improving Menstrual Health Among Women and Girls Using Community Engaged Intervention Development in Nepal**
SRG Action: [redacted]
Next Steps: **Visit https://grants.nih.gov/grants/next_steps.htm**
Human Subjects: **48-At time of award, restrictions will apply**
Animal Subjects: **10-No live vertebrate animals involved for competing appl.**
Gender: **1U-Both genders, scientifically unacceptable**
Minority: **5A-Only foreign subjects, scientifically acceptable**
Age: **1A-Children, Adults, Older Adults, scientifically acceptable**

Project Year	Direct Costs Requested	Estimated Total Cost
1	[redacted]	[redacted]
2	[redacted]	[redacted]
3	[redacted]	[redacted]
4	[redacted]	[redacted]
5	[redacted]	[redacted]
[redacted]	[redacted]	[redacted]
[redacted]	[redacted]	[redacted]

[redacted]

INCLUSION OF WOMEN PLAN UNACCEPTABLE
PROTECTION OF HUMAN SUBJECTS UNACCEPTABLE

RESUME AND SUMMARY OF DISCUSSION: This resubmission proposes mentorship and training to enhance [redacted] transition to an independent research career focused on improving women's reproductive health in low- and middle-income countries. The research project would review prior work on menstrual seclusion interventions, and co-design and pilot a menstrual seclusion intervention with Nepali communities. The panel agreed the candidate was outstanding, as demonstrated by their strong publication and fieldwork records, expertise in reproductive health, complementary skills in participatory methods and film making, and experience in the field site. They judged that the career development plan was comprehensive, addressed needed skills, and was aligned with goals. Reviewers found that the topic of menstrual health and stigma was important in Nepal and for women's global health. They identified strengths in the novel and rigorous approach, the co-design and human-centered design elements, the community advisory board, and the potential for the feasibility study to serve as a basis for a future R01 proposal. However, reviewers noted potential weaknesses in the realist synthesis aim due to scant nature of existing literature and insufficient methodological description. The panel assessed the mentors, environment, and institutional commitments to be strong, and highlighted the representation of Nepalese among the committed mentors and organizations, and excellent reference letters. They noted a weakness in the lack of mentors with expertise in realist reviews. The resubmission was deemed mostly responsive to prior critiques, though reviewers thought efforts to build quantitative skills could have been further boosted. In sum, the panel determined that due to the outstanding strengths of the application, the training and research would highly improve the candidate's potential for an independent scientific career in global reproductive health.

DESCRIPTION (provided by applicant): Despite being a natural physiological process, menstruation is often associated with health and safety challenges, especially in low-resource settings. In Nepal, 90% of women and girls follow at least one menstrual restriction. The most extreme is chhaupadi, a social-religious tradition in which women live in isolation during menstruation, exposing them to harmful health consequences. There is a dearth of evidence regarding effective approaches to address chhaupadi, which remains widespread. Research: This community-engaged study aims to rigorously review extant intervention evidence, as well as co-design and pilot a culturally relevant chhaupadi intervention to ultimately improve women's health in Nepal. The study aims are as follows: Aim 1 - Conduct a Realist Synthesis to elucidate the mechanisms of success or failure of chhaupadi interventions to date, including key-informant interviews (KIIs) (n=35). Aim 2 - Co-design a chhaupadi intervention with women (n=30) in three districts using participatory, human-centered design activities. The intervention will be refined via community meetings (n=6) and KIIs (n=25). Aim 3 - Pilot test the intervention in two matched communities (intervention and control, n=45). Surveys with standardized measures and focus group discussions (n=6) will be conducted to assess feasibility, acceptability, and changes in health attitudes and behaviors expected to be impacted by the intervention. Candidate: Dr. [redacted] is an Assistant Professor at [redacted] with extensive previous training in community-engaged research and women's reproductive health in Nepal. Training: Four training goals will support [redacted] trajectory toward becoming an independent investigator: 1) Acquire skills in Realist Synthesis to identify underlying causal mechanisms and reasons for intervention success or failure; 2) develop expertise in intervention development and assessment, as well as implementation science; 3) attain new skills in clinical trial design applicable to low- and middle-income countries (LMICs); and 4) gain essential skills in leadership, mentorship, grant writing, and study management. Mentors/Environment: [redacted] will be supported by an exceptional team of international and

multidisciplinary experts including a diverse mentorship team, advisory board, consultants, and collaborating organizations in Nepal. Her mentorship team has expertise in essential content and methodological areas such as intervention development, evaluation, clinical trials, and statistical analyses. Her comprehensive career development plan includes substantial mentorship, didactic training, and experiential learning at [redacted] (US) and [redacted] (Nepal). By successfully completing these rigorous career development and research plans, [redacted] will be fully prepared to transition into a successful independent global health researcher and interventionist who harnesses community-engaged approaches to improve women's reproductive health in LMICs.

PUBLIC HEALTH RELEVANCE: Harmful menstrual practices, such as chhaupadi (menstrual seclusion) in Nepal, are an urgent threat to women's health, wellbeing, and development. This study will investigate underlying mechanisms of promising aspects of chhaupadi interventions to date and build upon these findings to co-design and pilot test a context- specific intervention in partnership with community members. In the short-term, outcomes from this proposal will advance the field by resulting in 1) a pilot tested intervention that can be further tested in a future randomized controlled trial, 2) a refined approach for engaging communities in sustainable and contextually appropriate intervention co-design that is expected to be applicable to other low- and middle-income country contexts, and 3) in the longer-term support public health efforts aiming to reduce the negative health effects of harmful menstrual practices on women in Nepal.

CRITIQUE [redacted]

Candidate: [redacted]
Career Development Plan: [redacted]
Research Plan: [redacted]
Mentors, Co-Mentor(s), Consultant(s), Collaborator(s): [redacted]
Environment & Institutional Commitment to the Candidate: [redacted]

Overall Impact: The candidate is an assistant professor in public health with the goal of becoming an interventionist specialized in women's health in LMIC settings, and has an impressive track record of publication and scholarly activity as well as field research. Overcoming menstrual health and stigma has implications for Nepal but also other areas of women's reproductive health. The career development plan will address the skills needed in realist synthesis and intervention design/evaluation to complement her current background, and to provide the platform for leadership and management to transition to independence.

1. Candidate:

Strengths

- Demonstrated commitment to reproductive health in LMICs via a range of field research experiences.
- Strong publication record, including existing mentorship activities.
- Has previously shown innovation in participatory research methods, including the development of an original collaborative film-based methodology piloted in Nepal.
- Excellent letters of reference.

Weaknesses

- None noted by reviewer.

2. Career Development Plan:

Strengths

- Excellent detailed listing of relevant coursework.
- Is clearly directed towards the career goals.
- Provides a solid basis for qualitative analysis.

Weaknesses

- None noted by reviewer.

3. Research Plan:

Strengths

- Strong co-design elements developed by the applicant themselves.
- Includes a novel and rigorous human centered approach.
- Appropriate feasibility study.

Weaknesses

- As noted by the candidate, the approach to the realist synthesis will very likely need to broaden beyond chhaupadi.
- Unclear if the Beck Anxiety scale is the appropriate measures to use for mental health.
- Timeline may be overly ambitious.

4. Mentor(s), Co-Mentor(s), Consultant(s), Collaborator(s):

Strengths

- Excellent team of mentors.

Weaknesses

- None noted by reviewer.

5. Environment and Institutional Commitment to the Candidate:

Strengths

- Extremely strong.

Weaknesses

- None noted by reviewer.

Study Timeline:

Strengths

- Study timeline is appropriate for the activities planned.

Weaknesses

- None noted by reviewer.

Protections for Human Subjects:
Acceptable Risks and Adequate Protections
Data and Safety Monitoring Plan (Applicable for Clinical Trials Only):
Acceptable

Inclusion Plans:

- Sex/Gender: Distribution not justified scientifically.
- Race/Ethnicity: Distribution justified scientifically.
- For NIH-Defined Phase III trials, Plans for valid design and analysis:
- Inclusion/Exclusion Based on Age: Distribution justified scientifically.
- No position articulated to justify regarding the inclusion/exclusion of men (in the roles of prospective partners/heads of family) or transgendered individuals.

Vertebrate Animals:
Not Applicable (No Vertebrate Animals)

Biohazards:
Not Applicable (No Biohazards)

Resubmission:

Resource Sharing Plans:
Not Applicable (No Relevant Resources)

Training in the Responsible Conduct of Research:
Acceptable
Comments on Format (Required):
Comments on Subject Matter (Required):
Comments on Faculty Participation (Required):
Comments on Duration (Required):
Comments on Frequency (Required):

Budget and Period of Support:
Recommend as Requested

CRITIQUE [redacted]

Candidate: [redacted]
Career Development Plan: [redacted]
Research Plan: [redacted]
Mentors, Co-Mentor(s), Consultant(s), Collaborator(s): [redacted]
Environment & Institutional Commitment to the Candidate: [redacted]

Overall Impact: This is a well-crafted and comprehensive proposal with clearly defined goals and a variety of diverse activities aimed at enhancing the candidate's career development in the area of women's reproductive health in LMICs. The candidate has been working on the practice of *chhaupadi,* and its negative impacts on women's health for some time, and with many of the key mentors mentioned in this application. The candidate has previously conducted qualitative research on this subject in Nepal, and is in a position to use this experience and data to inform Aims 1 and 2 of the proposed K-award research project. The research approach in this resubmitted proposal is compelling, as it aims to rigorously review extant intervention evidence, as well as co-design and pilot a culturally relevant *chhaupadi* intervention with communities. Although Aim 3 the study is not powered to show intervention effectiveness, it seems likely that the resultant data will form a solid foundation for a subsequent R01 application. The mentorship team is an impressive multidisciplinary group, with good Nepalese representation, and the inclusion of an Advisory Board is a positive point. The institutions involved in the application appear to provide a positive supporting environment for the candidate's activities, particularly as they already have an existing track record of collaboration with the candidate/mentors on this particular topic. Overall, the proposed career development and research plan seems highly likely to enhance the candidate's potential for a productive, independent scientific research career in women's reproductive health in LMICs, and more generally in research on traditional practices with negative impacts on women's health and well-being.

1. Candidate:

Strengths

- The candidate has a general long-term career goal to become an independent global health researcher who designs community-engaged interventions to improve women's reproductive health in LMICs, but has been focusing very specifically on menstrual health in Nepal throughout doctoral and post-doctoral work.
- Candidate has been highly productive (in terms of publications and involvement in grants) since obtaining PhD [redacted]
- Candidate has already conducted qualitative research in Nepal relevant to/supportive of the K-award research topic, with members of mentorship team and local organizations cited in the current proposal, and has learned the Nepali language.

Weaknesses

- Minor point: this resubmission aims to respond to reviewer comments on candidate's acknowledged limited quantitative training, and does so but only to a small extent, or at least the details of the capacity strengthening in statistics is not spelled out (see Mentorship, below).

2. Career Development Plan:

Strengths

- The candidate has identified an impressive array of capacity-strengthening activities (coursework, mentorship, conferences) relevant to the research proposed in the K-award proposal and consistent with the candidate's long-term career goals.
- A cross-cutting objective wisely looks beyond what is needed specifically for the K-award research to basic skills needed as an academic researcher in global health (leadership, mentorship, grant writing, study management).
- Some translation of information/training into practice is provided through meetings/activities with mentors.

Weaknesses

- The number of seminars, workshops and courses may raise a concern about workload (especially in year 2) as the design and implementation of research intervention is going on simultaneously. In some cases, it is not clear how long the courses (etc.) run and what is required from participants.
- Minor point: there appears to be no mentor involvement in the cross-cutting objective activities (only coursework/workshops, page 104) but interactions with mentorship team may help with the development of skills mentioned.

3. Research Plan:

Strengths

- Previous qualitative research findings have given the candidate important preliminary data to help direct intervention development.
- The process of evaluating past *chhaupadi* interventions, elucidating the mechanisms behind their success/failure, and consulting experts through interviews seems innovative and useful to help hone in on interventions with greater potential effectiveness in Aim 2.
- The community-based co-design process of potential *chhaupadi* interventions as extensively described in Table 3 (page 110) is creative and compelling.
- Exploratory outcome measures could give some idea of intervention impact and the data collected could help in the design of a future R01 study.

Weaknesses

- Realist Synthesis (RS) is central to the intervention development, and mentioned a number of times, but I could not see where this approach was described/explained in any detail.
- Given the contextual differences revealed in prior qualitative research regarding *chhaupadi* practices, it is hard to imagine one intervention being feasible/acceptable/effective to reduce harm in all situations, though the process of (co-) designing such interventions might be generalizable.

4. Mentor(s), Co-Mentor(s), Consultant(s), Collaborator(s):

Strengths

- The candidate has assembled an impressive mentorship team, including in-country Nepalese expertise, whose roles in the K-award project are clearly delineated.
- The candidate has been working with the main mentors [redacted] for a considerable amount of time. Both have significant experience in mentoring junior faculty.

- The plan includes hybrid mentorship meetings with the full mentorship team every 4 months and development/maintenance of the candidate's individual development plan (IDP): this seems a good way of keeping mentorship team members engaged and the candidate on track.
- The inclusion of an advisory board whose members have expertise in relevant aspects of research in Nepal, and who provide biannual feedback on research activities, could be very constructive.
- The comprehensive collective letter of support by core members of the mentorship team expresses great enthusiasm and commitment.

Weaknesses

- Minor point: there is little detail as to the mentorship in statistics to be provided by [redacted]; formal training is limited to the Fundamentals of Clinical Trials 3-course series at Pitt.

5. Environment and Institutional Commitment to the Candidate:

Strengths

- [redacted] is a highly resourced institution with longstanding experience and excellent track record in global health research. Its institutions and individual experts, both academic and administrative, are more than capable of providing adequate support for the candidate's research and training activities.
- [redacted] is far less known to this reviewer, but the candidate is likely to profit from interactions with faculty and students in the Nepal context, and [redacted] is ultimately the main resource from [redacted] of interest for this project.
- Two local organizations stand out as assets: [redacted] (to help with logistics) and the [redacted] (to help with input to intervention design and pilot testing).
- Strong letter of support from Nepalese collaborators/institutions who will also be involved in the Advisory Board.

Weaknesses

- Minor point: there is little description of how research administration functions in the Nepalese context, i.e. what permissions the candidate might need (also in communities) for the research to move forward. On the other hand, the candidate has conducted similar research there before, and has worked with the Nepalese Health Ethics Board.

Study Timeline:

Strengths

- The study timeline in itself appears to unfold reasonably over Aims 1-3 in the five years of the project, without significant overlap of activities.

Weaknesses

- Some concern about workload when study timeline is put together with the many career development activities. On the other hand, the productivity of the candidate since PhD graduation suggests the ability to manage a high workload.

Protections for Human Subjects:

Unacceptable Risks and/or Inadequate Protections

- Something only briefly touched on in the application are potential unintended consequences of research activities. Pilot testing of interventions involves changes to practices and norms that appear rooted in gender inequity, so it may be worth considering whether research activities can raise risks of gender-based violence (or other social harms). There are expert members in the mentorship team very well placed to assess this.

Data and Safety Monitoring Plan (Applicable for Clinical Trials Only):

Acceptable

- No DSMB involved, data safety and monitoring will be conducted by the candidate, with consultations with mentors when necessary.

Inclusion Plans:

- Sex/Gender: Distribution justified scientifically.
- Race/Ethnicity: Distribution justified scientifically.
- For NIH-Defined Phase III trials, Plans for valid design and analysis: Not applicable.
- Inclusion/Exclusion Based on Age: Distribution justified scientifically.

Vertebrate Animals:

Not Applicable (No Vertebrate Animals)

Biohazards:

Not Applicable (No Biohazards)

Resubmission:

- The candidate appears to have addressed the majority of reviewer comments adequately. Most of the comments focused on the research strategy, the responses to which appear to have led to a more coherent, detailed approach.

Resource Sharing Plans

Not Applicable (No Relevant Resources)

Training in the Responsible Conduct of Research:

Acceptable

Comments on Format (Required):

- There are various formats (in-person/online; seminar/workshop) and all are appropriate.

Comments on Subject Matter (Required):

- The subject matter is relevant, particularly the ethics in research training in Nepal.

Comments on Faculty Participation (Required):

- There is sufficient involvement of mentors in the candidate's RCR training, though it is doubtful that the 120 hours stated (p. 113) will be entirely focused on RCR.

Comments on Duration (Required):

- The amount of hours for RCR far exceeds NIH requirements. The candidate should consider scaling back somewhat. It seems redundant to do the CITI course and the Pitt RCR course, for example.

Comments on Frequency (Required):

- The frequency of RCR training thorough the course of the project far exceeds NIH requirements.

Budget and Period of Support:

Recommend as Requested

CRITIQUE [redacted]

Candidate: [redacted]
Career Development Plan: [redacted]
Research Plan: [redacted]
Mentors, Co-Mentor(s), Consultant(s), Collaborator(s): [redacted]
Environment & Institutional Commitment to the Candidate: [redacted]

Overall Impact: This resubmission is submitted by a PhD trained [redacted] and Assistant Professor at [redacted] who has significant experience conducting patient-oriented research to reduce morbidity and mortality related to women's reproductive health, with a focus on south/southeast Asia. She represents a productive record of contributions to science, publishing 14 articles in peer-reviewed journals, including 9 as first author; and obtaining 20 institutional grants, 3 foundation grants, and Fulbright/Boren fellowships. Her research to date has drawn on a variety of qualitative/mixed methods, community-engaged research, and human centered design. Her long-term career goal is to be a global health interventionist using community-engaged methods to develop, test, and deploy interventions for women's reproductive health in LMICs. To accomplish this goal she seeks additional training in realist reviews and intervention development/evaluation (intervention development, randomized trials, and implementation science). Paired with these training goals she proposes a realist systematic review of anti-*chhaupadi* (seclusion and banishment during periods of menstruation) interventions (Aim 1); using mixed methods and principles of human centered design to co-create, with Nepalese women and girls who practice *chhaupadi*, an anti-*chhaupadi* intervention (Aim 2); and pilot testing the intervention with 45 women and girls (n=30 treatment / n=15 control) in two communities (Aim 3).

The A0 application was not discussed. The reviewers identified concerns in the research plan, including the potential public health significance of the proposed research, and a need for additional detail about the methods used. With respect to the training plan, the reviewers also noted overlap in the proposed training with the candidate's preexisting training. The A1 resubmission is responsive to the concerns raised: the career development plan has been significantly overhauled, with discarded training in areas in which the candidate already has expertise (HCD and SNA) and new emphasis on training in areas in which the candidate needs training (realist reviews, intervention development, and implementation science); and the research plan contains appropriate detail needed to assess the potential viability of the hypothetical intervention. The revised significance paragraph makes clear that this practice

continues despite being criminalized in 2017. Minor weaknesses do not detract from the assessment that the proposed training and research aims are likely to have significant public health impact.

1. Candidate:

Strengths

- The candidate has a broad-based training background in many areas that will benefit her intended career trajectory to become a global health interventionist using community-engaged methods to develop, test, and deploy interventions for women's reproductive health in LMICs.
- The candidate has published 4 articles in peer-reviewed journals specifically in the Nepal context.
- The candidate's limited publication record supporting mentees is not considered a weakness, given that this proposed K01 is a mentored research application and significant mentorship of other ESIs is not an expectation at this career stage.

Weaknesses

- None noted by reviewer.

2. Career Development Plan:

Strengths

- The career development plan proposes comprehensive training in realist reviews, intervention development/implementation science, and clinical trial design.
- The revised application jettisons the proposals to train in areas where she already has significant expertise (SNA and HCD).
- The revised application appropriately places the hypothetical R01 submission in years 4-5.

Weaknesses

- Minor: This reviewer would have expected that the candidate's institution would provide a suitable environment for training, but with the exception of the realist review training (which I would not expect to be offered at any US institution) and the [redacted] RCT series, nearly all of the training is obtained through external short courses.

3. Research Plan:

Strengths

- The revised application makes clear that *chhaupadi* remains a significant public health issue despite its criminalization in 2017.
- The candidate proposes a comprehensive suite of research activities to assess the anti-*chhaupadi* intervention literature and to co-design/pilot test a new anti-*chhaupadi* intervention.
- While the exact nature of the intervention is undefined (because it is to be developed in Aim 2), the candidate provides sufficient detail about the possible range of components.

Weaknesses

- Minor: Acknowledging that the preliminary effectiveness estimates generated in Aim 3 are not formally intended to be a cluster RCT, there is no mention of potential violations of SUTVA. To the extent that there are any spillovers from one community to the other (either beneficial

spillovers from the treated individuals in community 1 to the untreated individuals in community 2; or negative spillovers from untreated individuals in community 2 to the treated individuals in community 1) it will tend to bias any benefits toward the null.

4. Mentor(s), Co-Mentor(s), Consultant(s), Collaborator(s):

Strengths

- The mentors and co-mentors have the requisite scientific expertise in the areas needed for the proposed research activities.
- If there are any areas in which the mentors/co-mentors have overlapping expertise, the specific contributions of each mentor are noted.

Weaknesses

- Major: [redacted] (co-mentor) is identified as the individual who will provide mentorship in realist reviews. However, as far as this reviewer understands, while [redacted] has significant expertise in adolescent partner violence and reproductive coercion, she does not have expertise in realist reviews. To this reviewer's knowledge, [redacted] has led or supervised a single published article adopting the "realist review" process [redacted]). An over inclusive PubMed search -- "[redacted] and (review[pt] or systematic review[pt])" -- identifies no additional realist review publications.
- Minor: With the exception of [redacted] the other mentors' track records in providing primary mentorship on K-series awards is unclear.

5. Environment and Institutional Commitment to the Candidate:

Strengths

- The institution and collaboration provide a rich setting to support the proposed training and research plans.

Weaknesses

- Minor: This reviewer would have expected that the candidate's institution would provide a suitable environment for training, but with the exception of the realist review training (which I would not expect to be offered at any US institution) and the [redacted] RCT series, nearly all of the training is obtained through external short courses.

Study Timeline:

Strengths

- The timeline is appropriate for the proposed training and research activities.
- The revised application appropriately places the hypothetical R01 submission in years 4-5.

Weaknesses

- None noted by reviewer.

Protections for Human Subjects:

Unacceptable Risks and/or Inadequate Protections

- The candidate identifies the potential, but minimal, risk of discomfort generated by discussion of sensitive topics and of confidentiality breaches. Another potential risk, not identified by the candidate, is the possibility that the anti-chhaupadi intervention has the inadvertent effect of stigmatizing the practice. While the reduction of some health risk behaviors has been historically achieved through a mix of deliberate and non-deliberate stigmatization and that this is viewed as a net positive (e.g., population reduction in smoking), on balance there is much literature to suggest that such stigmatization may have negative unintended consequences (cf. Brewis & Wutich, "Lazy crazy & disgusting: stigma and the undoing of global health").

Data and Safety Monitoring Plan (Applicable for Clinical Trials Only):

Unacceptable

- A data and safety monitoring plan is provided, but it addresses only the minimal risks noted by the candidate and not the potential for stigmatization as described above.

Inclusion Plans:

- Sex/Gender: Distribution justified scientifically.
- Race/Ethnicity: Distribution justified scientifically.
- For NIH-Defined Phase III trials, Plans for valid design and analysis: Scientifically acceptable.
- Inclusion/Exclusion Based on Age: Distribution justified scientifically.

Vertebrate Animals:

Not Applicable (No Vertebrate Animals)

Biohazards:

Not Applicable (No Biohazards)

Resubmission:

- This A1 resubmission is responsive to the concerns raised by the reviewers: the career development plan has been significantly overhauled, with discarded training in areas in which the candidate already has expertise (HCD and SNA) and new emphasis on training in areas in which the candidate needs training (realist reviews, intervention development, and implementation science).

Resource Sharing Plans

Not Applicable (No Relevant Resources)

Training in the Responsible Conduct of Research:

Acceptable

Comments on Format (Required):

Comments on Subject Matter (Required):

Comments on Faculty Participation (Required):

Comments on Duration (Required):
Comments on Frequency (Required):

Select Agent Research:
Acceptable

Budget and Period of Support:
Recommend as Requested

THE FOLLOWING SECTIONS WERE PREPARED BY THE SCIENTIFIC REVIEW OFFICER TO SUMMARIZE THE OUTCOME OF DISCUSSIONS OF THE REVIEW COMMITTEE, OR REVIEWERS' WRITTEN CRITIQUES, ON THE FOLLOWING ISSUES:

PROTECTION OF HUMAN SUBJECTS: UNACCEPTABLE. The panel found that the application did not adequately address the potential unintended consequences of intervention (e.g., stigmatize those who continue to practice seclusion) and said that plans were needed to mitigate that risk.

INCLUSION OF WOMEN PLAN: UNACCEPTABLE. The panel found insufficient justification for the inclusion or exclusion of men or transgendered individuals.

INCLUSION OF MINORITIES PLAN: ACCEPTABLE

INCLUSION ACROSS THE LIFESPAN: ACCEPTABLE

COMMITTEE BUDGET RECOMMENDATIONS: The budget was recommended as requested.

Footnotes for [redacted]

NIH has modified its policy regarding the receipt of resubmissions (amended applications).See Guide Notice NOT-OD-18-197 at https://grants.nih.gov/grants/guide/notice-files/NOT-OD-18-197.html. The impact/priority score is calculated after discussion of an application by averaging the overall scores (1-9) given by all voting reviewers on the committee and multiplying by 10. The criterion scores are submitted prior to the meeting by the individual reviewers assigned to an application, and are not discussed specifically at the review meeting or calculated into the overall impact score. Some applications also receive a percentile ranking. For details on the review process, see http://grants.nih.gov/grants/peer_review_process.htm#scoring.

MEETING ROSTER

[redacted]

Notice of NIH Policy to All Applicants: Meeting rosters are provided for information purposes only. Applicant investigators and institutional officials must not communicate directly with study section members about an application before or after the review. Failure to observe this policy will create a serious breach of integrity in the peer review process, and may lead to actions outlined in NOT-OD-22-044 at https://grants.nih.gov/grants/guide/notice-files/NOT-OD-22-044.html, including removal of the application from immediate review.

CHAIRPERSON(S)

[redacted]

MEMBERS

[redacted], [redacted]

[redacted]

[redacted]

[redacted]

[redacted]

[redacted]

[redacted]

[redacted]

[redacted]

SCIENTIFIC REVIEW OFFICER

[redacted]

Consultants are required to absent themselves from the room during the review of any application if their presence would constitute or appear to constitute a conflict of interest.
